# Modulation of Tau Pathology in Alzheimer’s Disease by Dietary Bioactive Compounds

**DOI:** 10.3390/ijms25020831

**Published:** 2024-01-09

**Authors:** Huahua Shi, Yan Zhao

**Affiliations:** 1 Department of Bioengineering, Harbin Institute of Technology, Weihai 264209, China; 17s030067@stu.hit.edu.cn; 2 School of Chemistry and Chemical Engineering, Harbin Institute of Technology, Harbin 150001, China

**Keywords:** tau pathology, Alzheimer’s disease, dietary bioactive compounds

## Abstract

Tau is a microtubule-associated protein essential for microtubule assembly and stability in neurons. The abnormal intracellular accumulation of tau aggregates is a major characteristic of brains from patients with Alzheimer’s disease (AD) and other tauopathies. In AD, the presence of neurofibrillary tangles (NFTs), which is composed of hyperphosphorylated tau protein, is positively correlated with the severity of the cognitive decline. Evidence suggests that the accumulation and aggregation of tau cause synaptic dysfunction and neuronal degeneration. Thus, the prevention of abnormal tau phosphorylation and elimination of tau aggregates have been proposed as therapeutic strategies for AD. However, currently tau-targeting therapies for AD and other tauopathies are limited. A number of dietary bioactive compounds have been found to modulate the posttranslational modifications of tau, including phosphorylation, small ubiquitin-like modifier (SUMO) mediated modification (SUMOylation) and acetylation, as well as inhibit tau aggregation and/or promote tau degradation. The advantages of using these dietary components over synthetic substances in AD prevention and intervention are their safety and accessibility. This review summarizes the mechanisms leading to tau pathology in AD and highlights the effects of bioactive compounds on the hyperphosphorylation, aggregation and clearance of tau protein. The potential of using these bioactive compounds for AD prevention and intervention is also discussed.

## 1. Introduction

Tau is a microtubule-associated protein mainly expressed in neurons. The major function of tau protein is to promote microtubule assembly and stability [1]. In physiological conditions, the association of tau with microtubules is regulated by the phosphorylation of tau at specific residues [2]. However, the aberrant phosphorylation of tau protein in pathological processes can decrease its binding to microtubules and cause its self-association, resulting in the formation of toxic tau aggregates and the disruption of microtubule networks [2,3]. In addition to the phosphorylation, a number of other posttranslational modifications, such as small ubiquitin-like modifier (SUMO) mediated modification (SUMOylation), acetylation and ubiquitination, have been identified to modulate the function and aggregation of tau [1,4,5].

The abnormal aggregation of tau protein has been found in a group of neurodegenerative diseases known as tauopathies, including Alzheimer’s disease (AD), frontotemporal lobar degeneration (FTLD), progressive supranuclear palsy (PSP) and corticobasal degeneration (CBD) [6,7,8]. Among these tauopathies, the most studied condition is AD. The accumulation of intracellular neurofibrillary tangles (NFTs) composed of hyperphosphorylated tau protein, along with the extracellular deposition of senile plaques formed by β-amyloid (Aβ) and neuronal loss, is a major pathological characteristic of AD [9]. Studies have shown that the number of NFTs in the brains of AD patients is positively correlated with the severity of the disease, suggesting that the abnormal phosphorylation and aggregation of tau are closely associated with the cognitive decline in AD [10,11]. Thus, the prevention of abnormal tau phosphorylation and elimination of tau aggregates have been proposed as therapeutic strategies for AD treatment. However, currently tau-targeting therapies for AD and other tauopathies with clinical efficacy are very limited [12,13]. Tau-targeting therapies using antisense oligonucleotides, which cannot distinguish pathological and non-pathological tau, can affect the normal physiological function of tau, leading to unwanted consequences. Anti-tau immunotherapies may elicit adverse immune responses and their effectiveness is dependent on the choice of epitope. To achieve optimal efficacy, the antibodies need to target both extracellular and intracellular pathological forms of tau. Small-molecule drugs targeting the post-translational modification, aggregation or degradation of tau protein face similar challenges, such as off-target toxicity and poor brain and neuronal accessibility [14,15].

In the past few decades, numerous dietary components have been found to possess anti-tauopathy properties. For example, the supplementation of green tea polyphenol epigallocatechin-3-gallate (EGCG), curcumin or resveratrol has been shown to reduce tau hyperphosphorylation and ameliorate the cognitive impairment in AD animal models and clinical studies [16,17]. The consumption of these therapeutic bioactive compounds or foods rich in them may prevent the development of tau-related pathology, thus reducing the incidence or slowing down the progression of AD [18,19]. One clear advantage of dietary components over synthetic substances for AD prevention and intervention is that they can be consumed safely as part of a balanced diet [20]. This review summarizes the mechanisms leading to tau pathology in AD and highlights the effects of bioactive compounds on the hyperphosphorylation, aggregation and clearance of tau protein. The potential of using these bioactive compounds in AD prevention and intervention is also discussed.

## 2. Tauopathy in AD

### 2.1. The Gene and Function of Tau Protein

**Figure 1:**
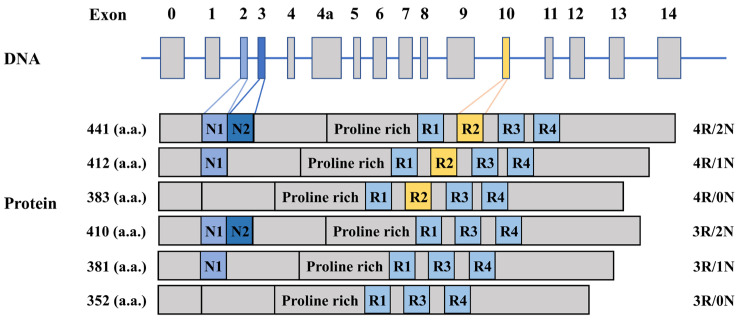
The human *tau* gene and tau isoforms. In adult human brain, *tau* gene encodes six tau isoforms 4R/2N, 4R/1N, 3R/2N, 4R/0N, 3R/1N and 3R/0N, which are generated from alternative splicing of exons 2, 3, and 10. “R” indicates microtubule-associated-binding repeat; “N” represents the N-terminal inserts.

The human *tau* gene is situated on the long arm of chromosome 17 at 17q21 [21]. It has been observed that a total of six predominant tau isoforms are expressed in adult human brain, which emerge from the alternative splicing of exons 2, 3 and 10 [1]. Exons 2 and 3 encode two different N-terminal domains. The presence or absence of both exons 2 and 3 results in 2 N or 0 N isoform, while the absence of either exons 2 or 3 results in 1 N isoform. Exon 10, which encodes the second microtubule-associated binding repeat, can be spliced in or out, generating tau with 4 or 3 microtubule-binding repeats, respectively [1,22]. Among the six tau isoforms generated, 3R/0N, 4R/0N, 3R/1N, 4R/1N, 3R/2N and 4R/2N, 4R/2N is the longest and 3R/0N is the shortest isoform, comprising 441 and 352 amino acids, respectively [22] (Figure 1). In mature human brain, the 3R and 4R tau isoforms are found in approximately equal molar ratios [23,24].

In neurons, tau is predominantly localized in axons, while it can also be detected in dendrites, though at much lower levels [25,26,27]. The microtubule-binding domains of tau and the flanking regions allow for the interaction of tau with both polymerized and unpolymerized tubulin, facilitating microtubule assembly, which forms the cytoskeletons within neurons and defines the neuronal morphology [28,29]. In addition to maintaining the morphology of neurons, the tau protein is critical for neuronal signaling, axonal transport, synaptic structure and function [30,31].

### 2.2. Post-Translational Modification of Tau Protein in Physiological and Pathological Conditions

Post-translational modifications alter the charge, hydrophobicity and conformation of a protein by adding chemical groups or protein units to specific residues of a target protein, thereby regulating protein function, protein–protein interactions and protein aggregation [32]. The post-translational modifications of tau, such as phosphorylation, acetylation, glycosylation and ubiquitination, play critical roles in regulating the interaction of tau with microtubules, as well as the localization, aggregation and degradation of tau [32,33,34]. Under physiological conditions, the post-translational modifications of the tau protein are important for modulating the function of tau [32]. For instance, tau phosphorylation at Ser262 and Ser356 in the microtubule-binding domains is necessary for neuronal outgrowth, while the tau phosphorylation of Ser/Thr Pro motifs in the regions proximal to microtubule-binding domains blocks neurite outgrowth [35]. In contrast, aberrant post-translational modifications alter the aggregation propensity and/or the function of tau, leading to tauopathy and disease development [36,37]. As an example, it is shown that the pseudophosphorylation of tau at Ser199/Ser202/Thr205 significantly impairs axonal transport in primary rat hippocampal neurons [31]. Cryo-electron microscopy and mass spectrometry analyses have revealed that the tau filaments from the brains of patients with AD and CBD are extensively post-translationally modified by phosphorylation, methylation, acetylation and ubiquitination, while the interplay between these post-translational modifications of tau protein influences the structure of the tau filaments [4]. Although the exact role of individual post-translational modifications remains undeciphered, there is no doubt that they are central in the regulation of the function and the aggregation propensity of tau.

#### 2.2.1. Phosphorylation of Tau

The 4R/2N tau from the human brain has 85 potential phosphorylation sites, including 45 serines, 35 threonines and 5 tyrosines [38]. Using phosphorylation-dependent antibodies against tau as well as mass spectrometry and sequence analyses, more than 31 phosphorylation sites have been identified to be associated with physiological functions. Three classes of protein kinases can phosphorylate tau: (1) proline-directed serine/threonine-protein kinases including glycogen synthase kinase-3 beta (GSK-3β), cyclin-dependent kinase-5 (CDK5) and mitogen-activated protein kinases (MAPKs); (2) non-proline-directed serine/threonine-protein kinases, such as microtubule affinity-regulating kinases (MARKs), Akt, AMP-activated protein kinase (AMPK) and Ca^2+^/calmodulin-dependent protein kinase II (CaMKII); and (3) tyrosine kinases such as Src, Fyn, Abl and Syk [5]. Tau can be dephosphorylated by a number of phosphatases including protein phosphatase 2A (PP2A), protein phosphatase 2B, protein phosphatase 1 (PP1) and protein phosphatase 5 [1,39]. The phosphorylation of tau is regulated by a balance between the above kinases and phosphatases, with GSK-3β and PP2A playing the most prominent roles [40,41]. GSK-3β is the major protein kinase that is associated with the excessive phosphorylation of tau, formation of neurofibrillary tangles and neuronal death [42,43]. In hippocampal neuronal cells, the activation of GSK-3β causes abnormal phosphorylation of tau at Thr181, Ser184, Ser262, Ser356 and Ser400, and induces the aggregation of the tau protein [44]. It has also been shown that the phosphorylation of tau at Thr231 by GSK-3β reduces the binding of tau to microtubules, resulting in the disruption of microtubule stability and axonal transport failure [45].

**Figure 2:**
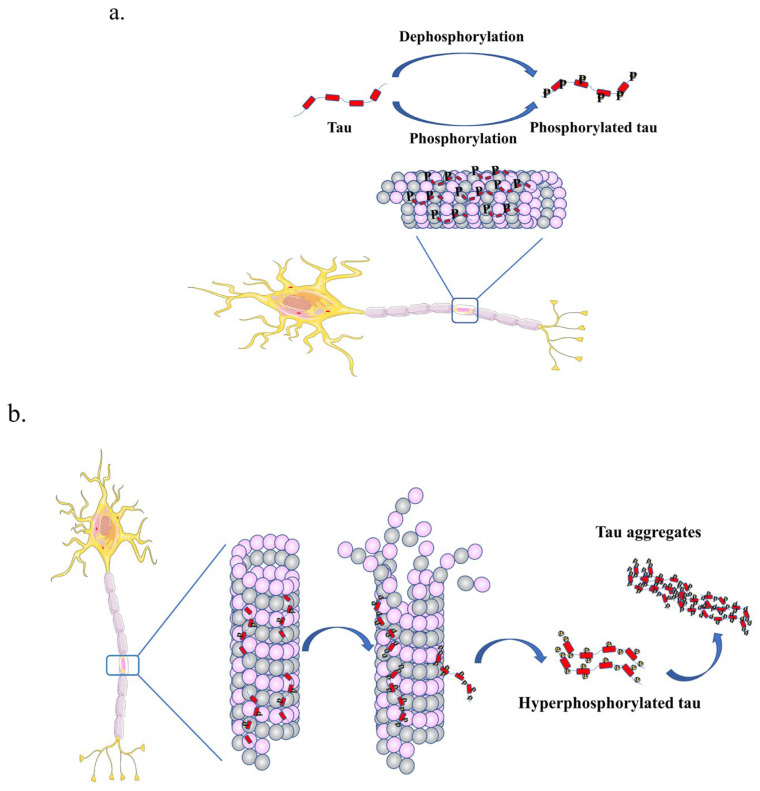
Tau phosphorylation in physiological condition and pathological state. (**a**) Tau regulates microtubule stability and dynamics in human neurons by directly binding to microtubules. The microtubule-binding repeats of tau protein bind at the inner face of the microtubules while the proline-rich region interacts with the surface of the microtubules. The interaction of tau with microtubules is regulated by phosphorylation via the concerted action of a variety of kinases and phosphatases. (**b**) In the pathological state, tau is hyperphosphorylated and no longer binds to microtubules, contributing to axonal dysfunction, and driving its oligomerization and aggregation into larger order insoluble fibrils.

Tau drives tubulin assembly into microtubules which form the cytoskeletons within neurons and define neuronal morphology [46,47,48]. Under normal physiological conditions, tau is phosphorylated at specific residues to regulate its association with microtubules (Figure 2a) [2,49]. In pathological states, specific sites on tau, for example, Ser262, Ser293, Ser324 and Ser356, localized in R1, R2, R3 and R4 domains, respectively, are aberrantly phosphorylated, reducing the association of tau protein with microtubules, increasing its propensity to self-associate and form toxic oligomeric species (Figure 2b) [44,50,51,52]. In AD brain, the highly phosphorylated tau protein loses its ability to bind to microtubules and aggregates to form paired helical filaments (PHFs), resulting in cytoskeleton abnormalities, axonal deficit and cell death [29,53].

#### 2.2.2. SUMOylation of Tau

SUMOylation, which adds SUMO to lysine residues of proteins via an isopeptide bond, is an important post-translational modification that regulates protein–protein interaction, intracellular trafficking, protein aggregation and degradation [54,55,56,57,58,59]. The overexpression of eGFP-labeled SUMO-1 in HEK293/tau cells, which stably express the longest isoform of human tau, significantly increases the phosphorylation of tau at Thr205, Ser214, Thr231, Ser262, Ser396 and Ser404, while the mutation of tau Lys340 to arginine (K340R) leads to the abolishment of tau hyperphosphorylation, suggesting that SUMOylation promotes the phosphorylation of tau [60]. On the other hand, the inhibition of PP2A significantly increases the immunoreactivity of SUMO-1 that is co-stained with the phosphorylated tau [60]. Furthermore, co-immunoprecipitation reveals a correlative tau hyperphosphorylation with an elevated tau SUMOylation after treatment with okadaic acid, a selective protein phosphatase inhibitor, suggesting that tau hyperphosphorylation enhances its SUMOylation [60]. Together, these data demonstrate that the SUMOylation and phosphorylation of tau promote each other. In addition, SUMO exhibits similarities to ubiquitin both structurally and biochemically in binding to substrate proteins [53,61,62]. Lysine residues are common targets for ubiquitination and SUMOylation. Studies have found that tau phosphorylation promotes its SUMOylation, while tau SUMOylation hinders its ubiquitination in AD brains, resulting in reduced tau degradation and increased tau aggregation [60]. These results strongly suggest that tau SUMOylation can promote the accumulation of tau aggregates by enhancing tau phosphorylation and inhibiting the ubiquitination-mediated tau degradation.

#### 2.2.3. Acetylation of Tau

The acetylation of tau has been shown to disengage tau from the microtubule and facilitate tau aggregation [63]. The immunohistochemical and biochemical studies of brains from tau transgenic mice and patients with AD as well as related tauopathies have shown that the acetylated tau is specifically associated with insoluble, thioflavin-positive tau aggregates [64]. The mass spectrometry analysis of post-mortem AD brains has demonstrated that Lys280 is the major site of tau acetylation [64]. Lys280 is located in the inter-repeat region of tau protein and has been identified as one of the three lysine residues most critical for modulating tau-microtubule interactions [65,66]. Increased tau acetylation on Lys280 can impair the interactions of tau with microtubules and increase the pools of cytosolic tau, which is subsequently used for the pathological aggregation of PHFs [64,66]. In addition, tau acetylation at other critical residues such as Lys174, Lys274 and Lys281 has been found to impair hippocampal long-term potentiation and promote AD-related synaptic defects and cognitive deficits [67,68]. 

In contrast, evidence suggests that the acetylation of tau within the KXGS motifs (Lys259, Lys290, Lys321, Lys353), which are conserved residues located in the microtubule-binding repeats of tau protein, inhibits tau aggregation [69]. It has been demonstrated that KXGS motifs in the tau protein are hypoacetylated and hyperphosphorylated in patients with AD, as well as in rTg4510 mouse models of progressive tauopathy [70]. The phosphorylation of serine residues in KXGS motifs (Ser262, Ser324 and Ser356), which reduces the binding of tau to microtubules and causes the destabilization of microtubules, is prevented by the acetylation of specific lysine residues in KXGS motifs [69,71]. Thus, targeted acetylation of these motifs may inhibit the phosphorylation and aggregation of tau protein, impeding the disease development. The acetylation of KXGS motifs can be mediated by p300 acetyltransferase [72] and deacetylated by histone deacetylase 6 (HDAC6) [70]. HDAC6 has been shown to mediate the deacetylation of KXGS motifs and increase tau aggregation in vitro. Consistently, the HDAC6 inhibitor treatment restores the acetylation of KXGS motifs, blocks the phosphorylation on this epitope and decreases tau polymerization [70]. Therefore, the selective inhibition of HDAC6 may lead to the reduction of tau aggregation and interference of the progression of tauopathies by increasing the acetylation of KXGS motifs on the tau protein.

These results implicate that, while acetylation modification is indeed important for regulating the polymerization and function of the tau protein, its effect relies on the specific sites where the acetylation occurs.

### 2.3. Tau Aggregation in Tauopathy

Electron microscopy analyses have identified several forms of polymerized structures of tau, including NFTs, PHFs and straight filaments (SFs), in the brains of AD patients [73,74,75]. The monomeric tau protein is highly soluble with little secondary structure. In contrast, β-sheet, α-helix and polyproline ΙΙ helical structures have been found in tau polymers [76]. There are 102 hydrophobic residues (Ala, Val, Iso, Leu, Met, Phe) and 85 putative phosphorylation sites in the 4R/2N tau molecule. The hydrophobic and/or ionic interactions among these amino acid residues are critical for the formation of secondary structures eventually leading to tau self-aggregation [1,76]. Two hexapeptide motifs in the 4R/0N tau, ^306^VQIVYK^311^ (PHF6) and ^275^VQIINK^280^ (PHF6*), exhibit the highest predicted β-structure potential within the tau sequence and are important for the assembly of tau into PHFs [77,78]. Moreover, the acetylation of the lysine residues within these two hexapeptides promotes the formation of β-sheet-enriched high-ordered oligomers [79].

Hyperphosphorylation introduces negative charges on the tau protein, altering electrostatic interactions between amino acid residues and causing conformational changes that may promote tau aggregation [80]. In addition, hyperphosphorylation disrupts the interaction of tau with microtubules, facilitating its binding to the unphosphorylated tau, thereby forming self-aggregates [81,82,83]. Evidence suggests that it is the phosphorylation of tau at specific sites rather than the overall phosphorylation state of tau that triggers tau aggregation. The combined phosphorylation at Ser202/Thr205/Ser208, together with the absence of phosphorylation at Ser262, yields a tau sample that readily forms fibers [84]. The phosphorylation of tau also leads to the unfolding of the “paper-clip” conformation of tau, resulting in the exposure of the N-terminal phosphatase-activating domain (PAD) of the tau protein, which is associated with the disruption of axonal transport [85]. This conformational change allows for the PAD to interact with PP1, which, in turn, activates GSK-3β via the dephosphorylation of Ser9 [86,87]. The activated GSK-3β then mediates the phosphorylation of tau at Thr231, which subsequently promotes the aggregation of tau [88].

The structure of tau oligomers, which are the intermediate forms of tau between the monomeric form and NFTs, is characterized by a secondary β-sheet containing 3 or 4 repeats of the microtubule-binding domain. After reaching a size greater than 20 nm, tau oligomers begin to aggregate and, as a result, form fibrillar forms [89,90]. Granular tau oligomers consisting of approximately 40 tau protein molecules have also been identified in the brain tissue of AD patients and are found to appear before the formation of PHFs [91,92]. Both in vitro and in vivo studies suggest that tau oligomers are the true toxic species and the best targets for anti-tau therapies [93]. Isolated tau oligomers, but not monomers or NFTs, induced memory impairments, synaptic dysfunction, and mitochondrial dysfunction when given intracerebrally to wild-type mice [94]. It is suggested that tau oligomers, like pathogenic seeds, are readily transferred from neuron to neuron propagating through the brain and induce neurodegeneration [95].

## 3. Clearance of Misfolded Tau by Protein Degradation System

Two major mechanisms that degrade the abnormal protein in cells are ubiquitin-proteasome system (UPS) and autophagy lysosomal pathway (ALP) [96,97]. Alterations of these proteolytic systems result in tau accumulation and often accompany pathological conditions [98,99]. The UPS degradation process includes two steps, substrate ubiquitination and substrate degradation. The ubiquitination is a key step in the selective degradation of protein quality control systems [100]. The identification of ubiquitin in PHFs in AD brains has led to the speculation that the UPS may have an important role in the degradation of tau aggregates [101]. The synaptic accumulation of phosphorylated tau in pre-and post-synaptic regions correlates with the reduction of UPS function in human AD brains [102]. Proteasome inhibitor treatment inhibits the degradation of the tau protein and leads to the pathological accumulation of tau in human neuroblastoma SH-SY5Y cells [103]. Ubiquitin C-terminal hydrolase L1 (UCH-L1) is an E3 ubiquitin ligase that is required for normal synaptic structure and function of hippocampal neurons [104]. In the brains of AD patients, UCH-L1 is co-localized with the hyperphosphorylated and abnormal ubiquitinated tau proteins, and the level of soluble UCH-L1 protein is inversely proportional to the number of NFTs [105]. The treatment of N2a cells with UCH-L1 inhibitor increases the phosphorylation of tau protein and decreases its microtubule-binding ability, suggesting that UCH-L1 may be important for the degradation of the hyperphosphorylated tau [106]. These results demonstrate that UPS dysfunction can cause an abnormal degradation of tau and promote the formation of NFTs.

Autophagy is a lysosomes-dependent degradation pathway that plays important roles in cell homeostasis by clearing damaged organelles, mutated proteins and protein aggregates [107,108]. In the early stage of AD, the accumulation of Aβ and tau induces autophagy to promote their removal [109,110]. Consistently, the hyperphosphorylated tau protein is colocalized with LC3B-II and p62, proteins critical for autophagic process, in brains from patients with AD [111]. It has been shown that lysosomal perturbation inhibits the clearance of tau in human neuroblastoma BE(2)-M17D cell line overexpressing tau isoform 4R/0N, causing the accumulation and aggregation of tau [112]. Likewise, the inhibition of the autophagic vacuole formation leads to a noticeable accumulation of tau in tau overexpressing M1C cells [112]. The autophagosome-lysosome fusion and degradation require the formation of ESCRT (endosomal sorting complex required for transport) complex [113]. Tau accumulation inhibits the expression of the IST1 factor associated with ESCRT-III and disrupts the ESCRT-III complex formation with repressed autophagosome-lysosome fusion [110]. Upregulating IST1 in human tau transgenic mice attenuates autophagy deficit while reducing tau aggregation and ameliorating the impairment of synaptic plasticity and cognitive functions [110]. The above evidence suggests that the autophagolysosomal pathway is critical for the degradation of tau aggregates in tauopathies.

Overall, these results suggest that both UPS and ALP are essential mechanisms for the clearance of the misfolded tau protein, the modulation of which may affect the progression of tau-related pathology. 

## 4. Modulation of Tau Pathology by Dietary Bioactive Compounds

Both the dysregulation of the post-translational modification of the tau protein and the alteration of the degradative mechanisms of misfolded tau contribute to the pathological accumulation of tau aggregates that correlates with the neurodegeneration in AD [10,11]. Accordingly, the inhibition of the abnormal post-translational modification of tau and the elimination of misfolded tau are considered to be important strategies for treating AD. Unfortunately, at present, the tau-targeting therapies for AD and other tauopathies remain limited [114]. Various dietary bioactive compounds have been reported to ameliorate tau pathology in cell and animal models. Here, we summarize their effects on the mechanisms involved in tau aggregation and degradation. 

### 4.1. Targeting Tau Post-Translational Modification by Bioactive Compounds

Studies have shown that a variety of bioactive compounds can affect the post-translational modification of tau. Curcumin, an antioxidant found in the rhizome of turmeric, *Curcuma longa* L. (Zingiberaceae), has anti-angiogenic, anti-inflammatory and neuroprotective properties [17,115]. Accumulating evidence suggests that the neuroprotective function of curcumin is associated with its modulation on tau phosphorylation. It has been shown that the long-term intake of low concentrations of curcumin delays the onset of AD while reducing tau phosphorylation and suppressing brain inflammation in amyloid precursor protein (APP)/presenilin-1 (PS1) transgenic AD mice [116]. In SH-SY5Y cells, curcumin pretreatment attenuates acrylamide-induced abnormal tau phosphorylation by suppressing PERK-eIF2α and the downstream GSK-3β signaling [117]. Molecular docking analyses have shown that curcumin can fit within the binding pocket of GSK-3β and is a selective inhibitor of GSK-3β [118]. Therefore, curcumin may suppress abnormal tau phosphorylation by directly interacting with GSK-3β or by the inhibition of the upstream PERK-eIF2α pathway.

It appears that GSK-3β is a common target of bioactive compounds. In both APP/PS1 and APP^NL-G-F^ transgenic mice, which carry three APP knock-in mutations associated with familial AD, marine carotenoid astaxanthin has been demonstrated to suppress GSK-3β activity and reduce tau hyperphosphorylation [119,120]. Resveratrol, a polyphenolic compound found in nuts and fruits, grapes in particular, has also been shown to exert neuroprotective effects by affecting tau post-translational modification. In age-accelerated mouse model SAMP8, the supplementation of resveratrol ameliorates the cognitive deficits while preventing the phosphorylation of tau at Ser396 in both the cortex and the hippocampus, possibly via a reduction in GSK-3β and CDK5 activity [121,122]. In addition, resveratrol treatment is found to decrease tau phosphorylation induced by various cellular toxicants including vanadate, cadmium and formaldehyde in cell and animal models [123,124,125]. Besides inhibiting the activation of protein kinases important in tau phosphorylation, such as GSK-3β and CaMKII, resveratrol has been shown to promote the dephosphorylation of the tau protein by elevating the activity of PP2A [123,124,125]. Similarly, by increasing the level of PP2A, the supplementation of rutin, an antioxidant with neuroprotective activities found widely in fruits, such as apricots, cherries, grapefruit and oranges [126,127,128], significantly reduced tau hyperphosphorylation in the brains of Tau-P301S mice overexpressing the P301S mutant form of human tau while rescuing synapse loss and preventing cognitive decline [129]. 

The above evidence demonstrates that natural bioactive substances can reduce the hyperphosphorylation and aggregation of tau protein by regulating the activities of major phosphokinases and phosphatases that determine tau phosphorylation, such as GSK-3β and PP2A. In addition to phosphorylation, dietary bioactive compounds have been demonstrated to affect the aggregation and degradation of tau through regulating tau SUMOylation and acetylation. The activation of c-Jun N-terminal kinase (JNK) and the elevation of SUMOylation have been found to enhance each other during oxidative stress [130,131]. In SH-SY5Y cells, the inhibition of H_2_O_2_-induced tau phosphorylation and cytotoxicity by curcumin is associated with the reduction of SUMOylation and JNK activation [131], though it remains unclear whether the reduction of SUMOylation is required for the inhibition of tau phosphorylation. Pretreatment with resveratrol has been shown to reduce tau hyperphosphorylation and acetylation while improving the cognitive performance in an aged postoperative cognitive dysfunction (POCD) rat model, possibly through restoring the expression of SIRT1, one of the main deacetylases regulating tau acetylation [132,133]. 

### 4.2. Targeting Tau Aggregation by Dietary Bioactive Compounds

Dietary bioactive compounds have been demonstrated to directly interact with tau and affect its aggregation. Thioflavin S staining and light scattering assay show that curcumin can inhibit the aggregation of 4R/0N tau in a concentration-dependent manner [134]. Images from atomic force microscopy suggest that curcumin significantly reduces the size of tau oligomers. Moreover, curcumin is able to disintegrate the preformed tau filaments [134]. Furthermore, results from far-UV circular dichroism spectroscopy and molecular dynamics simulations demonstrate that curcumin can disrupt the formation of local β-sheets and destabilize the tau protofibril structure, thus inhibiting the initial step of tau aggregation [134,135,136].

The microtubule-binding region (MTBR) of tau is prone to form β-sheet structures [137]. Molecular docking results have shown that curcumin may directly bind to the tau protein at MTBR. Analyses of the curcumin binding pocket of tau have revealed that Lys285, Asp194, Asp225 and Ser258 residues of tau can form hydrogen bonds with curcumin, while Val255, Val292, Leu195 and Val305 residues can form hydrophobic interactions with curcumin [134]. These interactions of curcumin with MTBR may prevent β-sheet formation in this region and eventually lead to the reduction of tau aggregation. Interestingly, it has been reported that curcumin and its analogs interact with tau oligomers by promoting the formation of higher-molecular-weight tau aggregates [138]. As the soluble, oligomeric tau proteins are likely the most toxic species [93], the aggregation of toxic tau oligomers by curcumin and its analogs may result in the formation of larger tau structures with a lower toxicity [138]. Additionally, the inhibitory effects of curcumin on tau amyloid fibril formation are more potent than its degradative products [139]. Given that curcumin is readily degraded under physiological conditions, formulations of curcumin with increased stability may enhance its potential therapeutic effects on tauopathies.

A few other dietary components have also been reported to be able to inhibit tau aggregation by directly binding to tau protein. Molecular dynamics simulation and in vitro aggregation assays have demonstrated that EGCG can inhibit tau aggregation by directly binding to tau at multiple sites; particularly, the interaction of EGCG with the postulated phosphorylated residues on tau protein may hinder the binding of kinases to these sites, therefore reducing tau phosphorylation [140,141]. In vitro aggregation assays have also revealed that rutin reduces tau aggregation and decreases the formation of tau fibrils, though detailed mechanisms need further investigation [129]. Myricetin, a flavonoid with antioxidant properties commonly found in vegetables, fruits, berries and nuts [142], as well as its glucosidic form, myricitrin, can slow the aggregation of tau induced by a liquid-liquid phase separation of the tau protein [143]. Molecular dynamics simulations have shown that myricetin can push the β-sheets apart, leading to a loosely packed structure where two of the four β-sheets dissociate, thus inhibiting the fibril formation of tau [144]. Grape seed proanthocyanidins (GSPs) are another group of bioactive compounds found to inhibit tau aggregation. Results from thioflavin S staining and transmission electron microscopy show that GSPs efficiently inhibit the aggregation of the repeat domain of tau protein (tau-RD) induced by heparin in a concentration-dependent manner [145]. In addition, GSPs significantly disassemble the pre-formed fibrils containing tau-RD. Further investigation with circular dichroism spectroscopy indicates that the binding of GSPs to tau disrupts the formation of β-sheets. Molecular dynamics simulations have suggested that GSPs can tightly bind to tau-RD via hydrogen bonds and hydrophobic interactions. Specifically, GSPs are predicted to interact with Tyr310 of tau-RD, which is a key residue in β-sheet structures and the π-π stacking of fibrillar architecture [145,146]. Therefore, the binding of GSPs to tau-RD may interfere with the intermolecular interactions of tau fibrils, thus reducing tau aggregation [145].

By inhibiting tau aggregation and disrupting the existing tau aggregates, these dietary bioactive components may be useful in the prevention and intervention of tauopathies such as AD.

### 4.3. Targeting Tau Degradation by Dietary Bioactive Compounds

Besides affecting tau aggregation, dietary bioactive compounds can modulate the degradation of misfolded tau. A number of dietary compounds have been demonstrated to reduce tau aggregates by enhancing ALP. It is shown that resveratrol treatment can rescue lead-induced neuronal autophagic dysfunction in both in vivo and in vitro models, thus preventing the accumulation of phosphorylated tau and Aβ [147]. Transcription factor EB (TFEB) is a master regulator of ALP [148,149], the activation of which has been shown to enhance lysosomal degradation of APP [150] and tau [151]. A curcumin analog named C1 directly binds to TFEB and promotes TFEB-mediated autophagy and lysosome biogenesis, while reducing the levels of tau aggregates in both P301S and 3×Tg-AD mouse models [152,153]. Additionally, myricetin has been shown to reduce tau aggregates and suppress tau toxicity in SH-SY5Y cells via inhibiting mTOR pathway and activating ATG5-dependent tau autophagy [143].

Dietary bioactive compounds have been reported to reduce misfolded tau by promoting UPS as well. Resveratrol supplementation reduces the presence of Aβ and tau pathology in the hippocampi of 3×Tg-AD mice, while elevating protein ubiquitination, increasing the levels of proteasome 20S core subunits and enhancing trypsin-like proteasomal activity [154]. Tanshinone IIA (Tan IIA) is one of the most abundant phenanthrenequinone compounds isolated from the roots of Salvia miltiorrhiza, a medicinal herb that has been used as a food supplement [155]. Treatment with Tan IIA increases the accumulation of polyubiquitinated tau and induces the proteasomal degradation of tau in HEK293 cells overexpressing human tau and primary neuron cells from 3×Tg-AD mice [156]. Interestingly, the increased clearance of misfolded tau by EGCG and resveratrol has been both associated with the elevation of the multifunction adaptor proteins p62 [157,158], which can directly bind to polyubiquitinated tau and target it for degradation by both autophagy and the proteasome [159,160], though further investigations are required to understand the detailed mechanisms.

Molecular chaperones play important roles in regulating protein homeostasis by promoting proper protein folding and targeting misfolded proteins for lysosomal and UPS-dependent degradation [161,162]. In aged mice overexpressing human tau, curcumin treatment restores the reduction of molecular chaperones heat shock protein (HSP) 90 and heat shock cognate protein (HSC) 70/HSP70 in membrane-enriched fractions while decreasing the soluble tau dimers. The elevation of HSPs might promote the clearance of misfolded tau and subsequently correct the pathological behavioral and synaptic deficits induced by tau accumulation [136]. Protopine, an isoquinoline alkaloid found in several medicinal plants such as Corydalis spp. and Fumariaspp [163], is an effective anti-tau agent that enhances memory functions in AD models [164]. Protopine treatment elevates the levels of HSC70/HSP70 and enhances the chaperone activity of HSP90 by acetylating HSP90 via reducing the binding of HDAC6 to HSP90, thereby facilitating the recruitment of HSPs and subsequently increasing the degradation of pathological tau [164]. In addition to enhancing the acetylation of HSP90 and expression of HSC70, protopine derivative bromo-protopine, a better HDAC6 inhibitor in comparison with the parent compound, has been shown to increase the expression of lysosomal-associated membrane protein type 2A, a receptor of chaperone-mediated autophagy, thus promoting tau degradation via chaperone-mediated autophagy in AD models [165].

In summary, dietary bioactive compounds may enhance the clearance of misfolded tau via multiple mechanisms. Nevertheless, by promoting the degradation of aberrant tau, treatments with dietary bioactive compounds may attenuate tau-related pathology in neurodegenerative diseases like AD.

## 5. Conclusions

**Figure 3:**
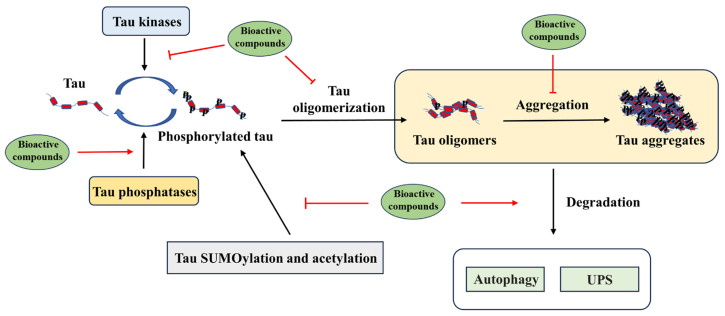
Possible protective mechanisms of bioactive compounds against tau pathology in AD.

Tau misfolding and aggregation lead to the formation of NFTs, which have long been considered as one of the main pathological hallmarks for a number of neurodegenerative diseases known as tauopathies, including AD. Clinical trials have suggested that tau-targeted therapies may be more effective than Aβ-targeted therapies in patients who already have neurodegenerative symptoms [3]. A variety of dietary bioactive compounds have been reported to significantly inhibit tau aggregation as well as promote tau depolymerization in vivo and in vitro [166,167,168]. The underlying mechanisms by which these bioactive compounds reduce tau aggregates include decreasing tau phosphorylation, promoting tau degradation and/or inhibiting tau aggregation (Figure 3). With potential therapeutic effects and minimal side effects, the supplementation of these bioactive compounds provides a promising approach for the prevention and intervention of tau-related pathology.

Nevertheless, several obstacles have to be overcome before the realization of AD therapies using dietary bioactive compounds. First, the in vivo solubility and absorption as well as their brain accessibility need to be further optimized to improve their bioavailability. New delivery methods incorporated with nanotechnology, which have been shown to greatly improve the access of these neuroprotective compounds to the central nervous system [169,170], may be used in the future. A combination of the natural products with synthetic drugs may achieve better therapeutic effects. As the molecular actions of dietary bioactive compounds against tau pathology remains elusive, more preclinical experiments are required to fully understand the underlying mechanisms. Lastly, and most importantly, clinical studies need to be performed to evaluate the efficacy and safety of these bioactive compounds in AD therapies.

## Data Availability

Data sharing not applicable. No new data were created or analyzed in this study.
